# Neurocognitive insights on conceptual knowledge and its breakdown

**DOI:** 10.1098/rstb.2012.0392

**Published:** 2014-01-19

**Authors:** Matthew A. Lambon Ralph

**Affiliations:** Neuroscience and Aphasia Research Unit, School of Psychological Sciences, University of Manchester, Zochonis Building, Brunswick Street, Manchester M13 9PL, UK

**Keywords:** semantic cognition, conceptual knowledge, semantic dementia, anterior temporal lobe, semantic memory

## Abstract

Conceptual knowledge reflects our multi-modal ‘semantic database’. As such, it brings meaning to all verbal and non-verbal stimuli, is the foundation for verbal and non-verbal expression and provides the basis for computing appropriate semantic generalizations. Multiple disciplines (e.g. philosophy, cognitive science, cognitive neuroscience and behavioural neurology) have striven to answer the questions of how concepts are formed, how they are represented in the brain and how they break down differentially in various neurological patient groups. A long-standing and prominent hypothesis is that concepts are distilled from our multi-modal verbal and non-verbal experience such that sensation in one modality (e.g. the smell of an apple) not only activates the intramodality long-term knowledge, but also reactivates the relevant intermodality information about that item (i.e. all the things you know about and can do with an apple). This multi-modal view of conceptualization fits with contemporary functional neuroimaging studies that observe systematic variation of activation across different modality-specific association regions dependent on the conceptual category or type of information. A second vein of interdisciplinary work argues, however, that even a smorgasbord of multi-modal features is insufficient to build coherent, generalizable concepts. Instead, an additional process or intermediate representation is required. Recent multidisciplinary work, which combines neuropsychology, neuroscience and computational models, offers evidence that conceptualization follows from a combination of modality-specific sources of information plus a transmodal ‘hub’ representational system that is supported primarily by regions within the anterior temporal lobe, bilaterally.

## 1. Introduction

Semantic cognition refers to a collection of interactive cognitive mechanisms that support semantically derived behaviours. We use our semantic or conceptual knowledge not only for verbal comprehension but also when we initiate language production (the purpose of receptive and expressive communication is, after all, the transfer of meaning from the speaker/sender to the listener/receiver). In addition, our considerable database of semantic knowledge is crucial in the non-verbal domain, both receptively (identification of non-verbal stimuli necessitates the transformation of sensation to meaning) and expressively (drawing and other expressive arts are based on the transmission of meaning, whilst effective object use requires semantic knowledge of each implement).

Semantic cognition can be decomposed into three interactive principal components underpinned by separable neural networks: (i) semantic entry/exit, i.e. translation between sensation/motor representations and semantic knowledge; (ii) the long-term representation of concepts/semantic memory; and (iii) ‘semantic control’—mechanisms that interact with our vast quantity of semantic knowledge in order to generate time- and context-appropriate behaviour [1,2]. Every semantic task (receptive or expressive) requires a variable combination of all three components. Consequently, when any one of them is compromised (after neurological damage or transient brain stimulation), participants will fail in semantic assessments though the quality of their impairment will vary.

This review is focused primarily upon semantic representation—that is the nature of coherent concepts, how they are represented and their neural basis. A brief detour into the nature of semantic entry/exit and control provides important information not only with regard to what each of these principal components of semantic cognition is, but also what semantic representation is not. In advance, however, it is worth underlining the observation that these three principal components have to be highly interactive in order to support semantic activities. Specifically, variation in efficiency within each system (either because the stimuli/concepts/contexts are inherently challenging or because a component has become compromised) will lead to automatic up- or downregulation of contributions from the other components. For example, the uncertainty that follows from noisy stimuli can be compensated by upregulating the bidirectional interaction with meaning (i.e. the semantic representations) or context. Likewise, there will be variable involvement of the three components depending on the nature and demands of the task or concept (e.g. for a formal exploration of this issue with respect to concrete and abstract concepts, see Hoffman *et al*. [3]).

Dedicated cognitive and neural machinery is taken up with semantic entry, i.e. reception of sensation and its translation into meaning, and also with semantic exit that is the transformation of meaning into the motor sequences that allow us to express our knowledge to others (e.g. through speech, writing, drawing, etc.). Each sensory–motor domain requires modality-specific computations that are necessary for transformation of sensation and these are supported by different cortical and subcortical regions and pathways. These sensory-specific processes are observed not only in functional neuroimaging studies but also through the modality-specific disorders exhibited by some neurological patients. Lissauer ([4] Jackson translation) was one of the first researchers to note that within the visual domain, there is a clear separation of patients with damage to the primary visual machinery (generating ‘apperceptive’ agnosia) versus other patients with deficits in higher-order ‘semantic’ representations (‘associative’ agnosia). Parallel distinctions are found in the other sensory domains with regard both to intact function (as revealed by *in vivo* neuroimaging) and neuropsychological studies [5]. The crucial distinction between entry/exit processes and core semantic representation is reflected in the difference between patients with modality-specific agnosia or word deafness and those with damage to core semantic representations. Specifically, the former patients exhibit a modality-specific impairment of entry to meaning—thus, for example, word deaf patients have difficulty understanding heard words but can retrieve full information about the same concepts if they read the word or see a picture of the same item [6]. By contrast—as discussed in more detail below—other brain regions are ‘transmodal’ and, when damaged, patients exhibit multi-modal deficits reflecting impairment of ‘central’ semantic representations.

The triumvirate within semantic cognition is completed with semantic ‘control’—which refers to a collection of executive and working memory-related processes that manipulate the core semantic representations in order to generate time- and task-appropriate behaviours in both the verbal and non-verbal domains [7,8]. These processes are critical to semantic cognition in all modalities: we store a wealth of information about the meanings of words/objects but frequently only a subset of this knowledge is required for a task—indeed, other aspects of knowledge can actually be inappropriate and unhelpful. For example, playing a piano requires information about fine movements of the fingers to be retrieved, yet moving a piano across a room requires very different actions [9]. In everyday semantic activities as well as formal assessments of semantic knowledge, it is often necessary to accentuate subordinate meanings, non-dominant features and to suppress other aspects of the same meaning. This is true both in receptive tasks and in semantically driven production. For example, in speech production, the speaker needs to select the appropriate label (e.g. animal, pet, dog, springer spaniel or ‘Oliver’) in order to convey the correct level of semantic specificity for a given concept (known in speech production research as the hypernym problem: [10]). Finally, the critical aspects of meaning can also change for the same concept over time, not only in language but also in non-verbal behaviour, e.g. object use. Imagine, for example, the very different uses of the same knife in the task of making a cheese and chutney sandwich: packet *opening*, bread *cutting*, butter *spreading*, cheese *slicing*, chutney *scooping*, etc., all require different, specific aspects of the knife's properties (and ways of holding and manipulating it) to be brought to the fore, one by one, whereas the most commonly listed property of *cutting* has to be inhibited, more often than not. Indeed, in the case of *scooping*, the canonical function of the knife has to be disregarded altogether and replaced by a substituted function in place of another object (spoon). In conclusion, the ability to regulate and shape conceptual information in all expressive and receptive modalities is critical to any adequate account of semantic cognition.

In the contemporary literature, the distinction between semantic representation and control was initially highlighted through a series of seminal functional magnetic resonance imaging (fMRI) studies with regard to the role of left inferior prefrontal cortex in semantic tasks [11–13]. Although there is an ongoing debate about the exact nature of the cognitive processes that are underpinned by this region (selection, inhibition, augmentation, etc.), all studies agree that the region does not store semantic knowledge *per se* but rather is important for its task-/context-specific manipulation. More recent studies have observed the same distinction in contrastive neuropsychological studies of patients with degradation of semantic representation (semantic dementia (SD)—the temporal lobe variant of frontotemporal dementia, see below) versus those with dysfunctional semantic control (semantic aphasia, see below: [1,14,15]). These neuropsychological studies along with parallel transcranial magnetic stimulation investigations [3,16,17] have highlighted a three-part neural network that seems to underpin the executive elements of semantic control (prefrontal cortex, posterior middle temporal lobe and the intraparietal sulcus, IPS). Although much of the functional neuroimaging literature has focused upon prefrontal regions, a recent meta-analysis of these fMRI studies found that the same triad of brain regions are implicated in semantic control as those found in patient and repetitive transcranial magnetic stimulation (rTMS) investigations [18]. While issues of control versus representation are active in the contemporary literature, closely related ideas are much older, stemming back through Luria to Goldstein and Head [14,19,20]. Indeed, Head's investigations of semantic aphasia highlighted some of the same key findings including the fact that the patients appeared to retain the core semantic knowledge but were unable to use it appropriately, that these semantic deficits overlapped with a broader range of information-processing (executive) impairments, and that this symptom complex arose from damage to the inferior parietal region.

## 2. Alternative views of conceptualization

### (a) Distributed-only accounts

What are conceptual representations and how does the brain encode them? Perhaps the most dominant hypothesis is that concepts are not stored as unitary representations in a specific brain region but rather reflect the mass action of multiple, modality-specific sources of information, each of which is coded in different cortical regions. While a considerable amount of the contemporary cognitive and cognitive neuroscience literature is still focused on this notion, the key ideas are not new and go back more than a century. Wernicke and Meynert (see [21]) were interested in how the brain formed and reactivated concepts—a process they referred to as ‘conceptualization’. Meynert and Wernicke's model of conceptualization made the following assumptions: (i) that the building blocks of concepts were modality-specific engrams (stores of information) localized to the cortical areas responsible for the corresponding sensory, motor or verbal domain; (ii) that these modality-specific engrams, in widespread brain regions, were fully interconnected; and (iii) that this web of connections was the basis of conceptualization—a specific concept being represented by the coactivation of all its associated engrams. For example, when tasting an apple, the taste-specific engram will automatically activate all of the other associated modality-linked engrams, enabling the brain to retrieve other knowledge concerning the object: its visual form, likely colour, name, presence of seeds, how it is peeled and so on. In this proposal, modality-specific engrams were located in particular brain regions, but conceptualization was not. Indeed, Wernicke–Meynert argued that—unlike forms of agnosia and aphasia—central disorders of conceptualization only occurred as a consequence of global brain damage (dementia), because only such widespread cortical damage would disrupt the engram reactivation process. As will be discussed below, this assumption turns out to be incorrect as there are indeed transmodal regions in the cortex, which when damaged or stimulated lead to multi-modal yet selective semantic impairment.

Direct descendants of these ideas are found in the modern literature though the language to describe them has been updated. In cognitive science, the idea of distributed, experience-dependant formulations of concepts is captured in the hypothesis of ‘embodied cognition’ [22], which can vary in form from weak to strong formulations [23]. Again, the key idea in these theories is that concepts reflect the mass action of multiple information sources that are experienced and encoded in each modality, separately. Considerable convergent evidence for this approach to conceptualization has come from functional neuroimaging studies [24,25], which have demonstrated that crucial sources of information for different types of concept (e.g. how things move, the sound they make, the way they look, the way we manipulate them, etc.) are activated in their respective modality-specific association cortices (i.e. human MT, auditory association cortex, ventral occipito-temporal visual association cortex, superior parietal/IPS, etc.)

### (b) Transmodal representation

As reviewed in more detail below, the need for and discussion of an additional transmodal representational system is found in multiple literatures, including philosophy and cognitive science (for careful discussion of related ideas, including ‘grounding by interaction’, see [26]). In addition, contemplation of the nature of different cortical regions and the impairments demonstrated by patients after damage to these areas also provides key insights about the foundation of semantic cognition. Moving beyond secondary modality-specific association cortices, there are intermediary ‘tertiary’ cortical areas that are not tied to any one particular modality—for which Luria [20] adopted the term ‘transmodal’ cortex. When viewed from the lateral surface, these form a horseshoe running from the IPS through the inferior parietal lobule, the middle temporal gyrus, anterior and inferior temporal region to various inferior and lateral prefrontal regions (areas that fall into the vascular ‘watershed’ between the middle cerebral, posterior cerebral and anterior communicating arteries). Such transmodal regions provide a neural opportunity not only for multiple sources of information to be merged into a coherent whole but also for other cognitive mechanisms to act on and influence processing in multiple modalities (although it should be noted here that the connectivity and thus exact functions will vary in graded ways across subregions within this transmodal horseshoe). Indeed, contemporary functional neuroimaging studies of multi-modal semantic activities highlight the very same network of regions [27,28] and, when this network is damaged, patients demonstrate multi-modal semantic impairment. Geschwind *et al.* [29], for example, reported a case of ‘isolation of the speech area’ following carbon monoxide poisoning (which tends to affect the vascular watershed regions and in this patient led to damage to most of the transmodal cortical areas) who was still able to repeat, generate non-propositional phrases and learn new songs but demonstrated significant semantic impairment. Other patient groups may exhibit modality-specific (e.g. verbal) comprehension deficits when lesions are isolated to modality-specific secondary association cortices (e.g. posterior superior temporal gyrus) but multi-modal impairments when the lesions are larger and encroach on the transmodal areas (e.g. posterior middle temporal and inferior parietal regions: [30,31]).

Cognitive functions vary across these transmodal regions. As noted above, functional neuroimaging, neuropsychological and rTMS studies all converge on the notion that prefrontal regions do not represent semantic knowledge *per se* but rather are crucial for task- and context-appropriate manipulation of the semantic database. There is less certainty about posterior temporal and inferior parietal contributions to multi-modal semantic processing. There are currently two main hypotheses about the functions of these posterior areas. A subset of patients with semantic aphasia have posterior temporoparietal damage, implicating these areas in semantic control [1,14] and indeed at least some functional neuroimaging studies and rTMS investigations have shown that posterior middle temporal gyrus (pMTG) and IPS are sensitive to the degree of semantic control required by the tasks [17,18]—which fits with the considerable evidence for IPS–prefrontal–pMTG interactions in a ‘multi-demand’ control network [32]. This pattern is also supported by recent comparative studies of chronic Wernicke's aphasia [31,33] which, in addition to a strong modality difference (spoken words < written words < picture comprehension), have found that the patients' multi-modal semantic impairment has the features of semantic aphasia (control deficits) rather than SD (i.e. representational degradation).

A second hypothesis is that some of these posterior areas are, instead, implicated in semantic representation rather than semantic control. Geschwind and co-workers, [29,34] for example, argued that the angular gyrus (AG) is ideally connected as a transmodal hub to code the meaning of words. In addition, contemporary functional neuroimaging studies have shown that the contrasts of concrete > abstract words, words > nonwords, etc., generate a significant difference in the AG [27,35,36]. Sophisticated neuropsychological studies of aphasic naming have recently associated ventral inferior parietal lobe (IPL) lesions with associative semantic errors [37], which could fit with the notion that the AG codes certain types of semantic information- or event-based relationships [35,38]. It is, of course, entirely possible that both hypotheses are correct such that different aspects of semantic cognition are tightly yet separately packed into the posterior temporal and IPL region. Future studies are required to reveal the nature and location of functions in this area. Indeed, there are many puzzles that remain. These include the facts that: the exact relationship between semantic control and representation has not been simultaneously mapped across this broader region; unlike other parts of the semantic network, the AG is often deactivated, yet differentially (less so for concrete than abstract, words than nonwords, etc.), which makes interpretation of its underlying function more complex [39]; and, also the observation that IPL regions are activated by numerous apparently different domains and tasks (including semantics, syntax, episodic memory, theory of mind, attention, phonology, praxis, etc.) and patients with lesions to this area often exhibit deficits in some or all of these domains [14,40].

### (c) The role of the anterior temporal lobe in semantic representation

Unlike the other regions of transmodal cortex noted above, until relatively recently the potential role of anterior temporal regions in semantic cognition has received much less attention. This is undoubtedly owing to multiple methodological limitations which amount to an absence of evidence about the anterior temporal lobe (ATL) rather than to evidence of absence. For example, although patients with SD were reported over a century ago [41], it was only modern neuroimaging techniques that allowed researchers to link the patients' semantic impairment with the underlying ATL damage. In addition, classical models of aphasia were primarily based upon patients with middle cerebral artery stroke which, owing to its vascular supply, is unlikely to damage the ATL region bilaterally, especially in its middle to ventral aspects [42]. Likewise, there is a related sampling bias within fMRI studies which, owing to various methodological issues (including limited field of view and magnetic inhomogeneities), have not consistently sampled activation in the middle and inferior ATL [43].

The case for the importance of transmodal representation in semantic memory and the role of the ATL in this aspect of semantic cognition has been heavily shaped by studies of patients with SD. SD, the temporal lobe variant of frontotemporal dementia, is associated with atrophy and hypometabolism of the ATL bilaterally. While the extent of the pathology spreads with progression, the distribution of atrophy is always most pronounced in the polar and ventrolateral aspects of the ATL [44–46] and is associated with the patients' increasing multi-modal semantic impairment. Owing to the relatively intensive and detailed neuropsychological studies of SD conducted over the past 20 years, we now know a considerable amount about the nature of the impairment in this patient group and, by extension, the nature of semantic representation and the functions of the ATL [47,48]. Some of the key characteristics of the patients' presentation—its *multi-modality*, *selectivity* and *progression*—were noted in the first significant modern neuropsychological study by Warrington [49]. She described three patients with progressive brain disease resulting in a range of multi-modal semantic deficits. Other aspects of the patients' cognition, including perceptual abilities and even other forms of memory (everyday episodic memory and short-term memory), were well preserved. Of course, semantic impairment can be found in a number of other ATL-related neurological conditions, including Alzheimer's disease, herpes simplex virus encephalitis, head injury and neurosurgery. Almost inevitably, however, the semantic impairment in these disorders is: (i) less pervasive than that observed in SD, and (ii) accompanied by other deficits affecting episodic or short-term memory, attention, executive and/or language processing—which makes the interpretation of impaired task performance harder given that one or more of the deficits beyond the semantic impairment itself may contribute to the observed task dysfunction. By contrast, the selective semantic degradation found in SD provides not only a key feature for differential diagnosis in the clinic [41] but also an unrivalled research opportunity to explore ATL-based contributions to semantic representation [49].

Detailed neuropsychological investigations have highlighted a plethora of insights about SD but two additional characteristics are important for this review. The first is that the semantic impairment is a graded phenomenon in which concepts and the boundaries between concepts gradually ‘dissolve’ or ‘dim’, rather than dropping out abruptly [50,51]. The second is that the degradation of concepts is multi-modal in nature, thereby leading to poor performance across all verbal and non-verbal domains, including words, objects, pictures, sounds, smells, and touch, etc., in receptive tasks and speech, writing, object use, drawing, etc., in expressive activities [48,52–57]. The simple, straightforward conclusion to draw from these studies of SD is that the ATL (bilaterally) plays a key representational role in conceptual knowledge and, following its transmodal nature, does this for all concepts, irrespective of the modality of input or output. Although the SD neuroanatomical and behavioural results are relatively clear, it is important not to rely solely upon a single source of information and, indeed, for some time the conclusions drawn about semantic memory and the ATL from SD alone were queried [24]. This is where convergent data from different methods are especially helpful, because the inherent yet non-overlapping weaknesses of each technique are ameliorated by using them conjunctively [3,58]. In recent years, there has been an accumulation of both functional neuroimaging (positron emission topography (PET), magnetoencephalography (MEG) and more recent distortion-corrected fMRI: [58,59–61]) and rTMS [62–65] studies of the left and right ATL regions in neurologically intact participants. These studies have found evidence that replicates and extends the SD patients' key characteristics, including multi-modality, bilaterality and selectivity. Thus, these studies have found that: (i) the ATL are activated for a range of semantic tasks, irrespective of the modality of input (e.g. words, pictures, sounds, etc.) as well as for concepts of different categories and levels of specificity (although activation and rTMS effects tend to be greater for specific-level concepts [63,66], they are also present for basic and domain-level distinctions: [61,65]); (ii) both left and right ATL areas are implicated in semantic processing; and (iii) the same areas do not appear to be involved in equally demanding non-semantic tasks (such as difficult number or novel visual stimulus judgements).

### (d) The hub-and-spoke model of semantic representation

The convergent evidence from the studies of SD patients, rTMS and functional neuroimaging can be understood on the basis that the transmodal ATL cortex plays a key and special role in the formation of semantic representations. Using a computational model, Rogers *et al.* [48] demonstrated that an interconnected central ‘hub’, which draws together modality-specific information, will behave as a transmodal representational system. The Rogers *et al.* ‘hub-and-spoke’ model is an extension of the Meynert–Wernicke ‘distributed-only’ framework. Information arising in each specific modality (e.g. the elephant's shape, colour, smell, form of movement, name, verbal descriptors, etc.) is coded in the corresponding specific cortical sensory or motor or language region. In this sense, the hub-and-spoke network as a whole *is* neuroanatomically widespread—as proposed originally by Meynert and Wernicke, and captured in modern ‘embodied cognition’ hypotheses. The information from the modality-specific regions, however, is fused together through an *additional* transmodal representation hub and, as a result, conceptualization reflects the joint action of the hub and the concept-relevant ‘spokes’ (i.e. the modality-specific sources of information that pertain to the target concept). The Rogers *et al.* model was trained to take a piece of modality-specific information (e.g. an outline of the elephant's visual form) as input and to reproduce the correct information across the remaining information layers (e.g. its colour or name or various things people might say about it, etc.) by propagating activation through the intermediate transmodal hub. Rogers *et al.* were able to demonstrate that simulated damage to these intermediate transmodal units of the trained model reproduced the core features of SD (and, although predating many of the functional neuroimaging and rTMS investigations, is consistent with the finding that the intact ATL regions are involved in normal, multi-modal semantic representation).

At the time the Rogers *et al.* [48] model was formulated, there was a relative paucity of information about the connectivity of ATL regions although the model assumed widespread connectivity from the transmodal hub to modality-specific association areas. Data from both comparative neurology [67] and recent human studies (using *in vivo* distortion-corrected probabilistic tractography: [68]) indicate that this is the case. The ATL region not only receives inputs posteriorly from visual and auditory-associated regions but also medio-anteriorly from olfactory and limbic-related frontotemporal regions. In addition, the pattern of graded intratemporal lobe connectivity would seem to be a direct foundation for both rostral and lateral convergence of information, from which a graded representational hub (centred on the ventrolateral ATL) could be formed [68].

Before considering in more detail why coherent concepts might require the addition of a transmodal hub, it might be useful to tackle and clarify five common, interlinked misconceptions/issues which have arisen about the hub-and-spoke hypothesis.

(1) *Hub-only* versus *hub-and-spoke*: our previous investigations and descriptions of the theory have been summarized and recast by some researchers as a strict contrast between an ATL *hub-only* representation of semantic knowledge versus the neuroanatomically distributed accounts associated with embodied cognition. This is, however, a false contrast. As made explicit through computational implementation, the Rogers *et al.* framework requires a combination of hub-*and*-spokes to generate multi-modal, coherent concepts. Although previous empirical studies have tended to focus on the importance of specific individual regions for semantic representation (in support of embodied or hub-and-spoke hypotheses), more recent studies have focused upon the combined roles of transmodal ATL and modality-specific regions in semantic processing [65]. For example, the transient nature of rTMS can be used to investigate and compare different neural regions within the same participants using the same test materials. A study used this approach to test and confirm that both the ATL hub- and modality-specific spokes do, indeed, contribute simultaneously to semantic representation. Consistent with the general lack of category differences in SD patients [69,70], ATL stimulation slowed healthy participants' ability to use semantic knowledge to generate the names of animals, manipulable and non-manipulable artefacts to an equal degree. By contrast, stimulation of the IPS region generated a category-specific pattern (slowing the naming of man-made items only) which was driven by a selective interference for manipulable items and is consistent with the hypothesis that this region (spoke) codes praxis information [65].
(2) *Amodal versus transmodal*: semantic aficionados may note that the term ‘transmodal’ has been used throughout this paper to describe the nature of the representations coded within the ATL hub. In most of our previous descriptions of the theory, we have tended to use the term ‘amodal’. This adjective was originally selected in order to emphasize the difference between the ‘spokes’, which are inherently linked to a modality-specific source, and the hub that acts as an intermediary between all modalities rather than belonging to any one in particular. Indeed, as discussed in the next section, there are good reasons to think that coherent concepts require some kind of modality-independent re-representation. For other researchers, however, ‘amodal’ is a loaded term and is associated with symbolic, non-embodied accounts of conceptualization [22]. As noted above, the hub-and-spoke theory is not an anti-embodied approach but rather emphasizes the need for *transmodal* distillation of multi-modal verbal and non-verbal experience as the basis for conceptualization. Furthermore, as discussed in more detail below (see ‘Future directions’), there is increasing evidence for a ‘graded’ hub where there is a softening of the boundaries between modality-specific and transmodal representation.
(3) *Differences in performance across tasks, modalities and items*: the notion of an amodal/transmodal ATL hub implies that its function should be inherently multi-modal in nature and that damage should result in multi-modal impairment. As reviewed above, there is clear convergent evidence for both of these predictions. In addition, some researchers have suggested that the level of involvement/impairment should be equivalent across different tasks and modalities. If all other things were equal then this would be correct but, as with much else in life, all other things are not equal. As demonstrated across an array of different computational models, performance on any one particular task is influenced not only by the core transmodal representations but also by the nature of the modality-specific resources/representations and, importantly, the nature of the mapping between them [48,71]. Thus, for example, the same level of damage to the semantic system will tend to generate a larger effect on verbal than picture-based tasks, because there is an arbitrary mapping between words and meaning but a quasi-systematic one from pictured objects to meaning. As well as the nature of the representational transformations required by the task, the pattern of physical connectivity between regions will also be crucial. Indeed, the impact of a strong left-hemisphere bias in connectivity from ATL to speech production mechanisms was explored in a neuroanatomically constrained model [55] and shown to be capable of explaining the more severe levels of anomia in patients with greater left than right ATL lesions (e.g. after unilateral resection: [72–74]) or in patients with asymmetrically left > right than right > left ATL damage, in the context of bilateral disease [55]. The same logic applies to variation of performance across different item types. SD performance is similar for different concepts only as long as the items are matched for key factors. In particular, SD patients exhibit strong effects of concept familiarity/word frequency [75,76], imageability [77] and typicality [78]. As long as these factors are controlled then the patients rarely, if ever, demonstrate different levels of performance for different categories of concept [69,70], other than a preservation of understanding number quantity [79,80] which is thought to depend on parietal rather than anterior temporal regions.
(4) *The ATL and specific concepts*: various studies have reported patients with poor identification of faces or other specific entities after ATL lesions [81,82] and SD patients exhibit a graded effect such that performance on specific concepts is always worse than basic and superordinate distinctions [49,83]. Similarly, ATL rTMS slows down naming of specific concepts and neuroimaging studies have identified ATL activation for the same task [63,66]. These and other findings have led some researchers to consider the possibility that the ATL regions are important for the representation for specific entities alone. An alternative hypothesis was demonstrated within the Rogers *et al.* hub-and-spoke model, which exhibited a graded specificity effect within a single representational system (i.e. specific and general concepts were not represented in separate systems). Instead, at all levels of impairment, the model exhibited a specificity gradient, because specific concepts require precise and fine reactivation of detailed information in order to distinguish one specific-level exemplar from another. By contrast, performance on basic and domain-level concepts is impaired but not to the same degree, because less semantic precision is required. This suggests that the ATL semantic regions are not dedicated to specific concepts alone but rather the specificity gradient simply reflects the level of semantic precision required by the task. This explanation is consistent with the fact that the effect of ATL rTMS or the deficits in patients with mild ATL-related semantic impairment is more apparent on specific concepts. It also suggests that with sufficient statistical power, it should be possible to observe engagement of the same areas, in processing of basic or domain-level semantic discriminations, which has been reported more recently in rTMS and distortion-corrected fMRI studies [61,65].
(5) *ATL* versus *temporal pole:* as noted previously, SD patients provided a major source of information about the role of ATL regions in multi-modal semantic processing. Given the progressive and graded nature of the underlying pathology, it is difficult to be sure about the anatomical boundaries of the areas within the ATL that are critical for semantic processing. Consequently, in assessing this region in other patient groups, functional neuroimaging, etc. studies have varied in terms of the area that different research groups have used as a region of interest: for example, adopting BA38 (temporal polar cortex), *y* < 0 in Montreal Neurological Institute (MNI) space, the boundary provided by the limen insula, and so on. These anatomical boundary variations are understandable but also will generate important differences and apparent inconsistencies in reported results. From more recent studies, it would appear that focusing on the most anterior, polar regions may lead to null results, whereas other ATL regions do seem to be heavily implicated in semantic processing. In particular, correlations between semantic dysfunction and glucose hypometabolism in SD point heavily towards a region of the ventral ATL caudal to the temporal pole (centred on *y* = −26; (even though both exhibit the most atrophy: [84]). Likewise, through the use of MEG, PET and distortion-corrected fMRI, functional neuroimaging studies are now also implicating a ventrolateral and not temporopolar region as a centre point of the ATL representational hub [28,58,59,61,85]. Given the angle of the temporal lobe in anterior commissure–posterior commissure aligned MNI space and the technical challenges of using gradient echo planar imaging fMRI in the ventrolateral ATL [43], regions of interest that focus on temporopolar cortex, *y* > 0, etc. are very unlikely to sample a key ATL region in semantic studies.

### (e) Why do we need a transmodal representation for coherent conceptualization?

The theoretical approach described here raises the important questions of: (i) what representation is coded or re-coded in the hub, and (ii) why does conceptualization necessitate an additional hub layer? As noted above, the hub-and-spoke model suggests that modality-specific association areas code information arising in each modality and these multiple sources are drawn together into coherent concepts through interactions with an ‘amodal/transmodal’ hub [86]. The computations required to achieve coherent concepts are non-trivial, because the modality-specific semantic features combine in complex, nonlinear ways. The relationship between ingredients and baked goods (e.g. pastries, breads, cakes, etc.) may be a useful analogy. It is always possible to consider each product (concept) and deconstruct it into a list of ingredients (features). Indeed, as per the classical semantic theories and contemporary embodied approaches, it is clear that the ingredients (features) are fundamental to the formation of each product (concept) in that if one of the features is missing (e.g. flour) is impossible to generate the concepts that contain it (e.g. most baked products). In addition to deconstructing the concepts into features, it is also important to consider the construction of concepts from features—given that this is the process that most theories assume underpins the formation of concepts. As with baking, although the presence of the correct ingredients is crucial, a pantry (brain) full of constituents (features) is not sufficient—we need a recipe (hub) as well. Indeed, as all novice bakers have experienced, following a recipe precisely is especially important as slight changes in the combination of ingredients leads to very different outcomes (e.g. a soggy brown lump versus a lemon-drizzle cake). This is because the ingredients (features) have a complex relationship with the product (concept). Indeed, this relationship is multi-dimensional and nonlinear. For example, using a fixed set of eight basic ingredients (flour, water, milk, eggs, butter, sugar, yeast and raising agent), it is possible to generate a cookbook full of baked products and, in some cases, extremely different outcomes start out from an identical ingredients list (e.g. croissants and buttered crumpets contain the very same ingredients). In short, the formation of coherent concepts (good cakes) requires *both* the modality-specific engrams/features (ingredients) and the transmodal hub representation (recipe).

The computational challenges that the formation of coherent concepts poses include the following.

(1) *Convergence of modality-specific and event-specific information*: sensory, motor and verbal modalities contribute variably to our semantic knowledge and this information does not necessarily arise at the same point in time. For example, <flying> and <laying eggs> are core aspects of most birds but we do not experience these features at the same time [87]. Thus, a mechanism is needed that systematically draws all this information together and does so in a time- and context-invariant fashion.
(2) *Features span different ranges of concepts*: both verbal and non-verbal features can extend in different, unrelated ways. Some features only apply to single entities, others extend across a group or patchy set of items while others are generally true of a whole class of concepts.
(3) *Complex set of nonlinear relationships*: as an extension of (2), it is clear that the multitude of features does not align and can sometimes be orthogonally, or nonlinearly related to each other. For example, vessels for pouring liquids can be grouped either by their specific function (e.g. for coffee, for tea, for wine, for watering plants, etc.) or by their material of construction (e.g. glass, porcelain, copper, plastic). Both types of feature are important for our specific interactions with each exemplar (e.g. when we want to make a drink versus when we want to clean the item without breaking it or to make it shiny), but these features do not have a one-to-one relationship (e.g. coffee pots can be made from a variety of different materials).
(4) *Surface similarities only a partial guide to meaning*: surface similarities (that is the statistics experienced in any one modality) are not a perfect guide to conceptual similarity and there are many feature ↔ concept opposing mappings. For example, some prominent aspects of an object are idiosyncratic—e.g. possessing an aesthetic design (e.g. a floral design may differentiate one specific teapot from the others but it is not a core defining feature of teapots *per se*); some aspects are common across the broad range of concepts but, as a consequence, finer categorization requires sensitivity to subtle variations; and, category membership has to be extended to exemplars that are superficially very different.
(5) *Semantic-based generalization to new or changing concepts*: another critical ability is semantic generalization—we often encounter new exemplars of an object (e.g. a new teapot) or existing examples change over time (e.g. your favourite teapot gets chipped, the decoration fades or you lose its lid) yet we easily and automatically generalize the knowledge about teapots to new or changing examples, even though we have never experienced these specific exemplars before.

These and many other related challenges have been highlighted before in philosophy [88] and cognitive science [89–91]. Wittgenstein, for example, noted that experience and use play a key role in concepts but that extracting shared meaning is not always possible on the basis of identifying a set of shared features. This included the observation that exemplars can vary in form (e.g. the different types of handles—break, switch, pump, crank—in a train driver's cabin; §12), or that knowledge can be generalized to new exemplars (e.g. knowledge of how to use the king in a set of chess pieces even if the design changes, §31) and that conceptually related items do not necessarily share any particular feature in common and thus cannot be defined in that way (e.g. games; §66). Following Wittgenstein's famous example, consider four category exemplars with the features {A,B,C}, {A,B,D}, {A,C,D} and {B,C,D}. No single feature defines them as a single group but instead they form a group through partial, overlapping features.

A potential solution comes in three parts: philosophical–cognitive science, computational and neuroanatomical. Rather than searching endlessly for defining attributes for each and every set of concepts, some philosophers [88, §67] and cognitive scientists have proposed that ‘coherent representations’ might follow if an additional computation or representation is added to our sensory–verbal experience (a full discussion of the various suggested solutions goes beyond the scope of this paper but broad and clear reviews can be found in [89,90]). This idea parallels a similar problem and approach found in computational modelling. In computational terms, this problem cannot be solved by a single layer of feature-coding units (a single layer perceptron), because the representations are not linearly separable. They can, however, be grouped properly if an additional ‘hub’ layer is added because this allows re-representation of the feature input. The ‘hub-and-spoke’ framework allows the formation of modality-invariant multi-dimensional representations that, through the cross-translation of information between modalities, code the higher-order statistical structure that is present in our transmodal experience of each entity. As such, the same core information is activated each time an entity is (re-)encountered even if different aspects occur in separate episodes (challenge 1); verbal and non-verbal features can be linked to different ranges of concept (challenge 2) whether or not the information is systematically or orthogonally related (challenge 3). As well as being able to code the partial similarity structure in specific domains, the modality-invariant representations add greater flexibility in order to deal with concepts that do not follow these surface similarities (challenge 4); and the system provides a mechanism for generalization to new or changing exemplars (challenge 5).

How does this modality-invariant representation system work and how does it break down in SD patients? By using an additional layer of representational units, computational models of semantic memory form a multi-dimensional space [48]. Through a gradual training process, each piece of sensory, verbal and motor information becomes associated with subregions of this space. A potentially useful analogy might be different kinds of geographical maps (though these are limited to two dimensions rather than the many found in semantic models). Each type of map (e.g. geological, political, linguistic, agricultural, etc.) codes the same chart/grid system with the presence or absence of each type of feature (e.g. mountainous regions, wheat-growing areas, etc.) that is found in that modality (type of map). This shared representational space (the grid system used in all maps) results in a multi-layered tapestry of information. Any specific location is then associated with (can reproduce) the information mapped to it (the name of the area, its geology, etc.) plus it can generate the likely information, for areas that have never been directly mapped, through interpolation (generalization to new examples).

By using many dimensions, it is possible to chart complex nonlinear regions—that is, map relationships between each feature and its associated concepts. In order to map a concept correctly in the high-dimensional, modality-invariant space, it is necessary to have a complex boundary. As this representational hub breaks down, two things are likely to happen: the boundary becomes fuzzy and is simplified, because there are fewer dimensions to code the boundary in. Two recent studies of SD patients investigated the effect that the degradation of the transmodal hub has on this aspect of conceptualization [86,92]. Irrespective of whether items were presented as words or pictures, the concept boundary in SD patients became increasingly dominated by superficial similarities (with reduced dimensions, this is an optimal solution in that classification accuracy can be maximized by aligning the boundary with the shared feature structure). The consequence, however, was that two types of mismapping occurred *simultaneously*—target exemplars that are superficially different to the average fall outside the changed boundary (undergeneralized) and non-exemplars that happen to share many features in common with the target become swallowed up (overgeneralized).

### (f) Future directions

Building on the important historical foundation provided by Wernicke, Meynert, Head and others, the past 20 years or so has seen considerable multidisciplinary research efforts applied to semantic cognition, including the nature of conceptualization and its neural basis. Inevitably, there are still many important issues and puzzles to be solved in future studies. A full exploration of them is beyond this paper, but four that relate to the nature of conceptual representations are discussed briefly below.

(1) *A graded representational hub*? Recent (distortion-corrected) neuroimaging studies have highlighted important variation of semantic function across the ATL region which seems to fit with the graded differences in connectivity and cytoarchitecture in this area [68,93]. To date, it would appear that the ventrolateral subregion may be the centrepoint of the graded ATL transmodal representational hub. Moving away from this point, there seem to be gradual shifts in the semantic function dependent upon the proximity/connectivity to different primary inputs [28,61]. These early, in-depth explorations of the ATL region are consistent with variants of the hub-and-spoke model that include graded variations of connectivity [94]. Future studies will be able to map the semantic functions of the ATL region in more detail and compare the results directly to the pattern of connectivity and cytoarchitecture in this area.
(2) *Unilateral versus bilateral ATL*: as noted previously, SD patients have bilateral (though often asymmetric) ATL atrophy indicating that both left and right regions may contribute to conceptualization. Key questions, therefore, are: (i) does semantic impairment require bilateral ATL damage and, if so, (ii) what are the roles of each ATL region? Recent studies that have explored semantic function in patients with unilateral ATL damage indicate that semantic performance is generally much better than that found in patients with bilateral ATL diseases but that, with more sensitive assessments, expressive and receptive semantic deficits can be observed [72,73,95]. These contemporary studies fit with the older comparative neurology literature which found evidence for multi-modal (visual and auditory) semantic impairment in primates after bilateral but not unilateral ATL resection [96,97], a pattern that was replicated in a rare human single-case neurosurgery study that was conducted soon after [98]. Further developments of the Rogers and co-workers [99] computational model have also replicated these clinical findings and provide some important clues as to why bilateral damage might always be much more disabling than unilateral lesions, even when volume of damage is controlled. Future studies are required to explore the contribution that each ATL makes to semantic representation, given that different hypotheses are already embraced in the current sparse literature. Implemented computational models of semantic representation have taken the stance that a unitary transmodal hub might be supported by a bilateral, interconnected ATL neural network, making the resultant system robust to damage [55,99], whereas various neuropsychological studies have indicated that there may be important variations across the hemispheres in terms of the modality of input/output or category of information [100–102].
(3) *Division of labour between the hub and spokes*: investigations of the hub-and-spoke hypothesis of conceptualization have focused primarily upon the comparison of representations within the hub versus spokes. The computational models of this hypothesis indicate that hub and spokes work in tandem to support semantic representation (see above). Future studies are required, however, to explore the division of labour across the two. It is, for example, possible to contemplate two extreme positions. The first is a ‘bottom up’ notion in which as much representation as possible is conducted in the modality-specific regions (because this is where the core sources of information are first encoded), and the transmodal hub is engaged only for fusion of information or where conceptual boundaries are hard to compute based on the modality-only sources of information. The second and opposite position is based on the idea that, having gone to the considerable computational effort of forming a transmodal hub, the individual redundant contributions from each modality can be downregulated. Indeed, it is possible that there may be variation in the division of labour between hub and spokes depending on the nature and demands of the task or types of concept (e.g. abstract versus concrete, atypical versus typical exemplars, specific versus general concepts). These issues are hard to explore through neuropsychological studies but other techniques, such as functional neuroimaging and TMS, licence cross-region comparison on a within-subjects basis.
(4) *The continued development of concepts*: much of the neuroscience literature on adult semantic cognition is based around studies of ‘stable’ semantic representations that have been acquired over a lifetime of experience. There is much less knowledge, however, of how concepts continue to evolve over time or even how new concepts are formed in adulthood. This is, perhaps, a slightly surprising state of affairs given that computational models have already demonstrated that the formation of new concepts or integration of new information into existing concepts has to overcome significant challenges (catastrophic interference: [103,104]). This is where greater dialogue between developmental and adult studies could be of great assistance. Indeed, there is a rich and informative literature on the formation and transformation of concepts through childhood. A greater interchange of techniques and methods between the two empirical domains could help us to understand the neural basis of initial concept development and transformation, and also would offer methods to study novel concept acquisition and the assimilation of new information into existing adult concepts.
